# Development of a Costimulatory Molecule Signature to Predict Prognosis, Immune Landscape, and Response to Immune Therapy for Hepatocellular Carcinoma

**DOI:** 10.1155/2022/8973721

**Published:** 2022-09-12

**Authors:** Yongjie Zhou, Xin Zhou, Qingxin Liu, Zihan Zhang, Wen Zhang, Jingqin Ma, Minjie Yang, Jiaze Yu, Jianjun Luo, Zhiping Yan

**Affiliations:** ^1^Department of Interventional Radiology, Zhongshan Hospital, Fudan University, Shanghai, China; ^2^Shanghai Institution of Medical Imaging, Shanghai, China; ^3^National Clinical Research Center for Interventional Medicine, Zhongshan Hospital, Fudan University, Shanghai, China; ^4^Center for Tumor Diagnosis and Therapy, Jinshan Hospital, Fudan University, Shanghai, China

## Abstract

This work was aimed at investigating the predictive value on prognosis, response to immunotherapy, and association with the immune landscape of costimulatory molecules in HCC patients. We acquired the clinicopathological information and gene expression of HCC patients from public available database (TCGA and GEO). The prognostic model in TCGA database was established with LASSO regression and Cox regression analysis. Through the Kyoto Encyclopedia of Genes and Genomes (KEGG) and Gene Ontology (GO) analysis, the enrichment analysis was implemented for analyzing the biological function and associated pathways. Immune microenvironment, immune escape, immune therapy, and tumor mutation were analyzed between both risk groups. TNFRSF4, the critical costimulatory molecule, was chosen for the in-depth investigation in vitro experiments. A novel risk signature based on 8 costimulatory molecules associated with prognosis was constructed from TCGA and proved in the database of GEO. The ROC and Kaplan-Meier curves confirmed that this risk model has good predictive accuracy. Our functional analysis demonstrated costimulatory molecular genes might associate with immune-related functions and pathways. Statistical differences were not shown between both groups, in the aspect of immune landscape, response to immune therapy, and tumor mutation. Knocking down TNFRSF4 expression significantly reduced the proliferation ability and increased the apoptosis ability. On the basis of the costimulatory molecule expression in HCC, a novel risk model was constructed and had an excellent value to predict prognosis, immune microenvironment, and response to immune therapy. TNFRSF4 was identified as an underlying oncogene in HCC and deserves further exploration.

## 1. Introduction

Hepatocellular carcinoma (HCC) was the most prevalent cancer and the third major cause of deaths associated with cancer around the world [1]. The most prevalent causes of HCC are nonalcoholic steatohepatitis, excessive consumption of alcohol, and chronic viral hepatitis (B and C) [2]. Surgery resection and ablation were recognized as curative therapies; however, the majority of HCC patients were classified in the intermediate to advanced stage at initial diagnosis. Despite great advances in diagnosis and treatments for HCC, the survival outcome for HCC is still unsatisfactory. The five-year survival rate was only 18%, because of recurrence and metastasis [3]. The characteristics of biological diversity and genomic heterogeneity further reduced the efficacy of the treatments for HCC [4]. Hence, figuring out the potential molecular mechanisms of HCC and exploring a novel treatment modality were significant.

Immunotherapy, especially immune checkpoint inhibitors (ICIs), has resulted in the revolutionary term of tumor therapy [5]. The introduction of blocking immunotherapy against the programmed death-ligand 1 (PD-L1)/programmed cell death protein 1 (PD-1) has given impressive results and prolonged the survival time of advanced HCC patients [6]. Regrettably, only a fraction of patients benefited from immunotherapy, because the immune-associated side effects also dampened the efficacy of immunotherapy.

The costimulatory molecule which activated T cells played a critical role in immunotherapy. The costimulatory molecule could identify the worthy antigenic stimuli for the immune system [7]. According to previous research, the B7-CD28 [8] together with tumor necrosis factor (TNF) family was constituted by costimulatory molecules [9]. The B7-CD28 family comprised 13 molecules, and the TNF family is composed of the TNF receptor superfamily (TNFRSF) and TNF ligand superfamily (TNFSF) containing 48 molecules. However, the effect of the costimulatory molecule on HCC carcinogenesis has not been elucidated.

In this work, a new prognostic signature with the costimulatory molecule-related genes of HCC patients from TCGA cohort was tested externally in the GEO. We in-depth explore the association of immune infiltration, immune microenvironment, immune escape, immune therapy, and tumor mutation with our prognostic model. The TNFRSF4 was selected to investigate biological function in vitro experiments.

## 2. Materials and Methods

### 2.1. Database

We received the expression profile of HCC tumor and normal specimen from TCGA database (https://http://portal.gdc.cancer.gov/repository), with relevant clinical information. The gene profile (GSE 27150) from GEO database was also downloaded to validate our risk model. The somatic mutation of patients with HCC was retrieved from TCGA database. The costimulatory molecule genes were determined from the earlier study, as displayed in Supplementary Table S1.

### 2.2. Identification of Differentially Costimulatory Molecule Genes and Tumor Classification

The expression information of costimulatory molecule-related genes was extracted from TCGA data. The differentially costimulatory molecule genes (DSMGs) were selected, and the criteria was FDR < 0.05 and |log2FC| ≥ 1, with the “limma” package in R. The network of protein-protein interaction (PPI) was established with STRING database (https://string-db.org/). The heat map of DSMGs was plotted by using the “heatmap” package, and we drew the volcano plot through the “ggplot2” package in R.

The consensus cluster analysis of expression profile with costimulatory molecule genes was performed by the “Consensus Cluster Plus” package with *K*-means method. The Kaplan-Meier curve was established for assessing the diverse cluster prognosis and in contrast to log-rank test.

### 2.3. Establishment and Validation of the Prognostic Signatures on the Basis of Costimulatory Molecule Genes

By using the HCC patients from TCGA cohort, we preliminarily selected the costimulatory molecule genes related with the prognosis with univariate Cox regression method. To avoid the overfitting offsets, we implemented the regression analysis of the least absolute shrinkage and selection operator (LASSO) to choose optimum coefficient through applying “glmnet” package in R. The *λ* value was determined with minimum criteria. Then, the risk scores for the HCC patients were calculated as the subsequent formula: risk score = (*Y* : expression profile of the gene; *X* : coefficient of the gene). We distributed the patients into low- and high-risk groups based on median risk score. The survival outcome was with log-rank and Kaplan-Meier survival plot test. For the prognostic model, its discriminative ability was determined through ROC curve. The correlation of patients from both groups was visualized through t-SNE analysis and PCA, with the “Seurat” package in R. We also identified the GSE 27150 data as the external validation cohort to test the predictive capability of such prognostic model. According to the above-mentioned risk score criterion, the patients' risk score from GSE 27150 was counted. In accordance with median risk score, the patients could also be classified as low- and high-risk groups. ROC curve, log-rank test, Kaplan-Meier survival, t-SNE analysis, and PCA were also implemented in GSE data.

### 2.4. Independent Prognostic Analysis and Functional Enrichment Analysis

The predicted values in GEO and TCGA data were chosen with multivariate and univariate Cox regression analysis. Utilizing the “forestplot” package in R, the forest plot was carried out. DSMGs were chosen between both groups, utilizing the “limma” R package. Based on these DSMGs, we explored the biological process together with the associated pathways with KEGG and GO analysis, using the “clusterProfiler” package.

### 2.5. Evaluation of Immune Landscape and Immune Therapy

Through “gsva” package, single sample gene set enrichment analysis (ssGSEA) was performed for investigating the immune cell proportion in tumor tissue, pathways related to immune. The ESTIMATE method was used to analyze tumor microenvironment. The expressions of immune checkpoint molecules (CD80, CD86, CD274, CD276, CTLA4, PDCD1, PDCD1LG2, and VTCN1) were compared between our two groups. The immunotherapy response was predicted with Tumor Immune Dysfunction and Exclusion (TIDE) algorithm using the web (http://tide.dfci.harvard.edu). TIDE score was calculated with exclusion score and dysfunction score and compared them between both groups.

### 2.6. Estimation of Tumor Mutation and Tumor Mutation Burden

We used the waterfall chart to visualize the landscape of tumor burden by using “maftools” package. We calculated the tumor mutation burden for each patient and compared them between both risk groups.

### 2.7. Cell Culture

From Cell Bank of Type Culture Collection (Chinese Academy of Sciences, Shanghai, China), human normal liver cell line L-02 and human HCC lines SKY-HEP-1, Huh-7, Li-7, and SNU-38 7 could be acquired. The lines L-02, Li-7, and SNU-387 were cultivated in RPMI-1640 medium (Biological Industries), and SKY-HEP-1 and Huh-7 cells were cultivated in DMEM medium (Gibco, Gaithersburg, MD, USA). Both the DMEM and RPMI-1640 medium were added with 1% penicillin–streptomycin mixture and 10% fetal bovine serum (Gibco). All of the cell lines were inoculated under a temperature of 37°C in 5% CO_2_ atmosphere.

### 2.8. RNA Extraction and qRT-PCR

In accordance with the direction (Omega, Norcross, GA, United States), the extraction of total RNA was conducted from the cell lines through utilizing the Trizol reagent. Applying the reaction reagents of the qRT-PCR kit (Tiangen, Beijing, China), the process of reverse transcription was carried out. The GAPDH was selected as a reference gene. The qPCR primer sequence is listed in Supplement Table S2.

### 2.9. Western Blot Analysis

Western blot was conducted according to the previous research. It is worth noting that antibodies applied in this work are as below: TNFRSF4 antibody (1 : 500, Proteintech, Shanghai), BAX antibody (1 : 5000, Proteintech, Shanghai), BCL2 antibody (1 : 1000, Proteintech, Shanghai), and GAPDH antibody (1 : 1000, Abcam, USA) were used for internal inference.

### 2.10. siRNA Transfection

The Li-7 and Huh-7 cells were transfected by siRNA-siTNFRSF4 and a negative control shRNA (siNC) according to the manufacturer's protocols. The siTNFRSF4 and siNC were made by Share-bio in shanghai. The RNA sequences for transfection are listed in Supplementary Table S2. qRT-PCR was utilized to test transfection efficiency.

### 2.11. Cell Proliferation and Apoptosis Assay

Colony formation and CCK-8 assay were conducted to test the cell proliferation ability of Li-7 and Huh-7 cells based on the instructions of the manufacturer. For the CCK-8 analysis, cells were inoculated in the plates (96-well) with 1000 cells/well density. Each well was added with CCK-8 solution (10 *μ*l, Beyotime, Shanghai, China) at a given time (24, 48, 72, and 96 hours). After incubation for 3 h, each well was detected at 450 nm with a spectrophotometer. For colony assay, 150 cells were incubated in every well of 6-well plates, and the medium was refreshed every three weeks. After two weeks, cells in each well were cleaned utilizing PBS, fixed by paraformaldehyde, and next stained through methylrosanilinium chloride solution. Finally, the number of cells in each well was counted. The apoptosis assay kit (KeyGEN BioTECH, Jiangsu) was performed for the apoptosis assay based on the protocols of the manufacturer.

### 2.12. Statistical Analysis

Spearman's or Pearson's association analysis was conducted to assess the correlation between both groups. The findings were displayed as frequencies and mean values ± standard deviation and next in comparison with Fisher's exact test, chi-square test, or independent *t*-test. The outcomes were statistically significant (*P* is less than 0.05). Visualization and data analysis were implemented with R software (4.0.4).

## 3. Result

### 3.1. Identification of Differentially Costimulatory Molecule Genes between Normal Tissues and HCC

**Figure 1:**
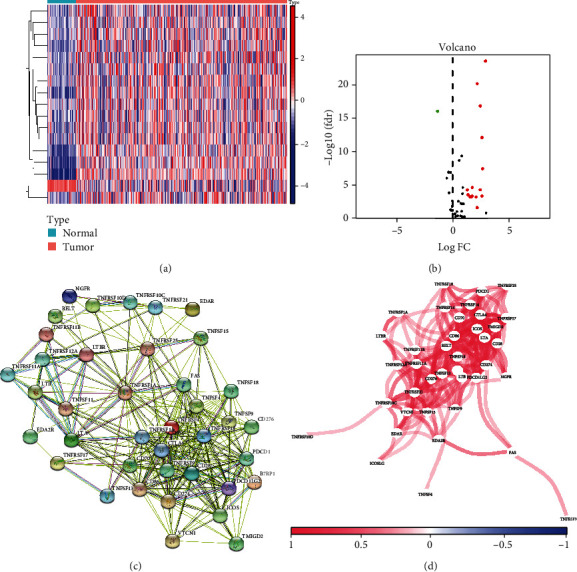
Identification of differentially costimulatory molecule genes. (a) Heat map and (b) volcano of differentially costimulatory molecule genes between tumor and normal tissues. (c) PPI network and (d) coexpression network of these genes.

The expression profile together with associated clinical information of HCC was gathered from TCGA LIHC data and GEO 27150 data. The clinicopathological characteristics of these patients with HCC are presented in Table 1. By comparing 60 costimulatory molecule gene expression levels from tumor tissue and normal tissues in TCGA LIHC data, we identified differentially costimulatory molecule genes which were presented in the heat map (Figure 1(a)). Among them, 16 genes were upregulated, while one gene was downregulated (Figure 1(b)). To further explore the connection between these differentially costimulatory molecule genes, we performed PPI analysis and coexpression network. The results showed that these hub genes deserved further investigation (Figures 1(c) and 1(d)).

### 3.2. Tumor Distribution on the Basis of Costimulatory Molecule Genes

**Figure 2:**
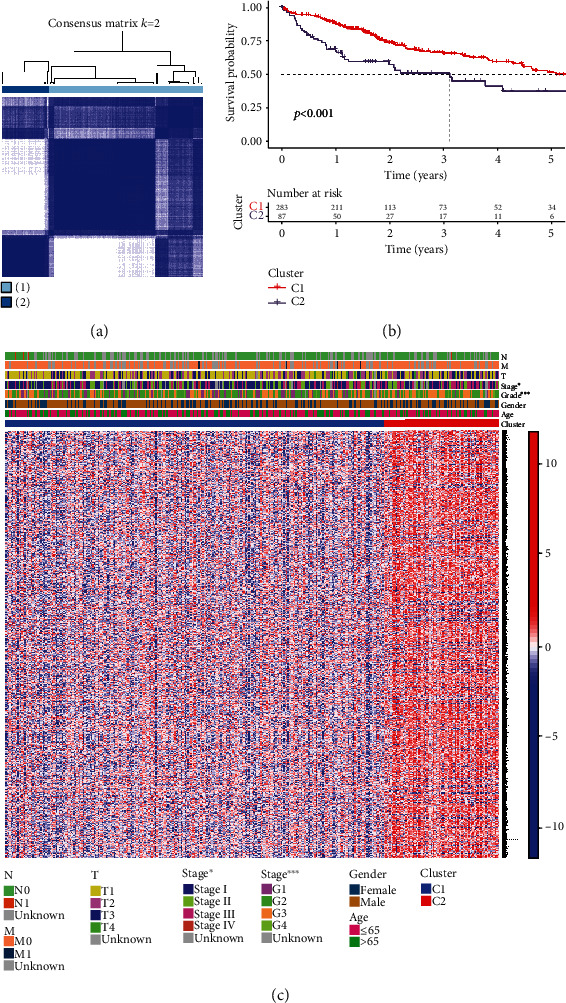
Tumor distribution on the basis of costimulatory molecule genes. (a) HCC patients could be classified as two clusters through employing consensus clustering (*k* = 2). (b) Kaplan-Meier survival for OS between two clusters. (c) Heat map of clinicopathological features and differentially costimulatory molecule gene expression levels between two clusters.

Consistent cluster analysis was implemented on HCC patients according to TCGA LIHC data for understanding the function of costimulatory molecular genes. By using the clustering variable (*k*), we eventually classified the HCC patients as two clusters (*k* = 2), as exhibited in Figure 2(a). The Kaplan-Meier survival analysis suggested that the prognosis of HCC patients in cluster 1 is greater in comparison with those in two clusters (Figure 2(b)). The differentially costimulatory molecule gene expression levels and clinicopathological characteristics in two clusters were presented in the heat map (Figure 2(c)), which showed that two clusters had significant differences in terms of stage and grade, and there was no statistical difference in T-N-M stage, sex, and age.

### 3.3. Establishment and Validation of Prognostic Signatures according to Costimulatory Molecule Genes

**Figure 3:**
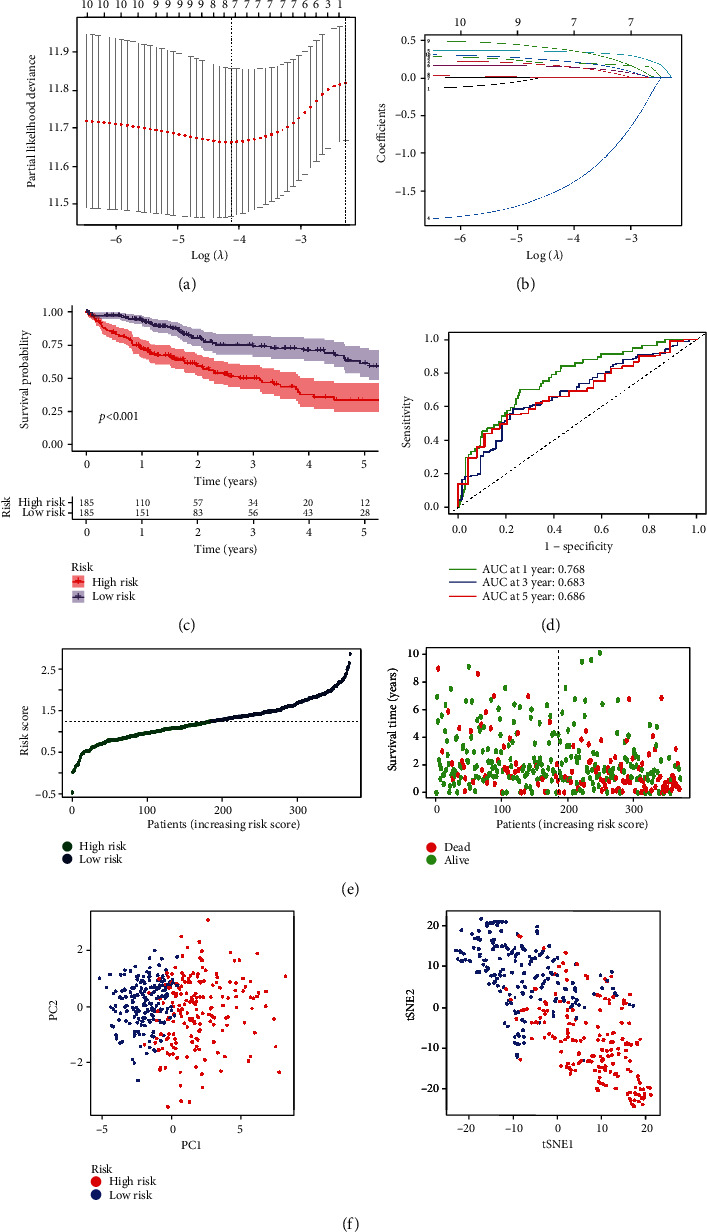
The establishment of prognostic signatures on the basis of costimulatory molecule genes. (a, b) LASSO regression was performed on prognostic costimulatory molecule genes preliminarily selected by univariate Cox regression, and eight genes were identified for constructing prognostic model. (c) Kaplan-Meier survival for OS of patients in both groups from TCGA data. (d) AUC of time-dependent ROC curves confirmed that our risk model has predictive property. (e) Distribution and correlation of survival time, survival status, and risk score of patients from TCGA data. (f) t-SNE and PCA analysis demonstrated the distribution of patients in two groups from TCGA data.

376 patients who meet our criteria were identified in our prognostic analysis. Firstly, we filtrated the prognostic costimulatory molecule genes (Supplementary Table S3), through utilizing the univariate Cox regression analysis. Subsequently, with LASSO regression analysis, we identified eight genes (LTBR, RELT, TMIGD2, TNFRSF11A, TNFRSF11B, TNFRSF21, TNFRSF4, and TNFSF4) to establish the prognostic model according to the optimal *λ* value (Figures 3(a) and 3(b)). We calculated the risk score as the following formula: 0.1497∗LTBR expression + 0.1945∗RELT expression + (−1.442)∗TMIGD2 expression + 0.3414∗TNFRSF11A expression + 0.1415∗TNFRSF11B expression + 0.0002∗TNFRSF21 expression + 0.3669∗TNFRSF4 expression + 0.2164∗TNFSF4 expression. The patients with HCC from TCGA data were distributed into high- and low-risk groups, in accordance with median risk score. The Kaplan-Meier curve displayed in comparison with low-risk patients, high-risk patients have a lower survival rate (Figure 3(c)). For the ROC curve, area under the curve (AUC) was 0.801, 0.751, and 0.720, for 1-, 2-, and 3-year survival, respectively (Figure 3(d)). Statistical differences of survival time and survival status of HCC patients were shown between the two groups (Figure 3(e)). Two groups were clustered in two relatively concentrated areas in t-SNE analysis and PCA (Figure 3(f)), which revealed the better discriminative value of this prognostic signature on the basis of expression values of costimulatory molecule genes.

**Figure 4:**
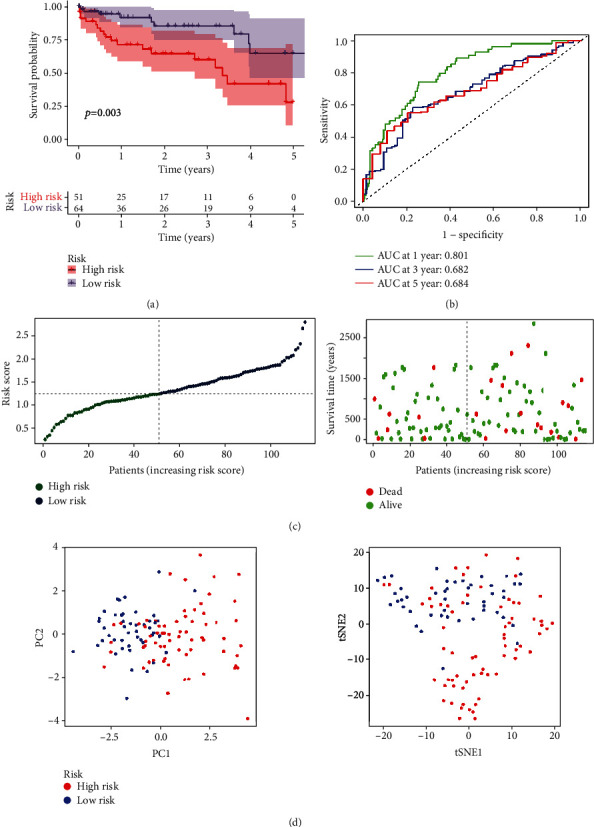
(a) Kaplan-Meier survival for OS of patients in both groups from the GEO data. (b) AUC of time-dependent ROC curves confirmed that our risk model has predictive property. (c) Distribution and correlation of survival time, survival status, and risk score of patients from GEO data. (d) t-SNE and PCA analysis confirmed the distribution of patients in both groups from GEO data.

114 HCC patients together with relevant clinical information were gathered from GEO to further assess the prognostic ability of such novel model. In accordance with above risk scoring formula, HCC patients were counted and classified as high- and low-risk groups. Kaplan-Meier survival curve displayed poor prognosis in high-risk patients (Figure 4(a)). For 1-, 2-, and 3-year survival, the ROC curve (AUC) were, respectively, 0.779, 0.681, and 0.752, which also indicated the excellent predictive values of this prognostic model (Figure 4(b)).  Figure 4(c) shows the survival time and status in two groups. Consistent with results from TCGA, patients in two groups from GEO were divided into two different orientations in the PCA and t-SNE analysis (Figure 4(d)).

### 3.4. Independent Prognostic Values of Risk Signature on the Basis of Costimulatory Molecule Genes

**Figure 5:**
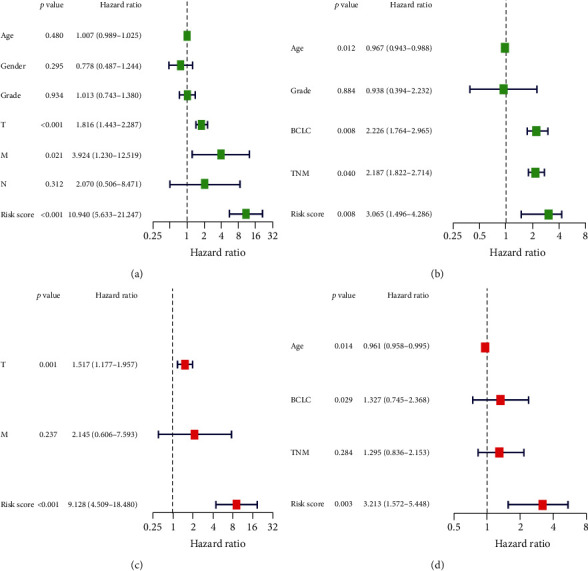
The multivariate and univariate Cox regression analysis for the risk score in both training and validation cohort. Univariate Cox analysis for patients from (a) TCGA cohort and (b) GEO cohort. Multivariate Cox analysis for patients from (c) TCGA cohort and (d) GEO cohort.

The multivariable and univariate Cox regression analysis was performed to in-depth assess predictive value of such risk model. The univariate Cox regression analysis suggested that HCC according to TCGA and GEO data, the risk score was a key predictor related to the patients' prognosis (HR = 10.940, 95%CI = 5.633–21.247, *P* < 0.001; HR = 3.065, 95%CI = 1.496–3.286, *P* = 0.008, respectively), as shown in Figures 5(a) and 5(b). After adjusting confounding factors, based on multivariable Cox regression analysis, there is a close association between risk score and poor prognosis (HR = 9.128, 95% CI: 4.509–18.480, *P* < 0.001; HR = 3.213, 95% CI: 1.5752–5.448, *P* = 0.003, respectively), as illustrated in Figures 5(c) and 5(d).

### 3.5. Biological Processes and Pathways Based on Risk Model

**Figure 6:**
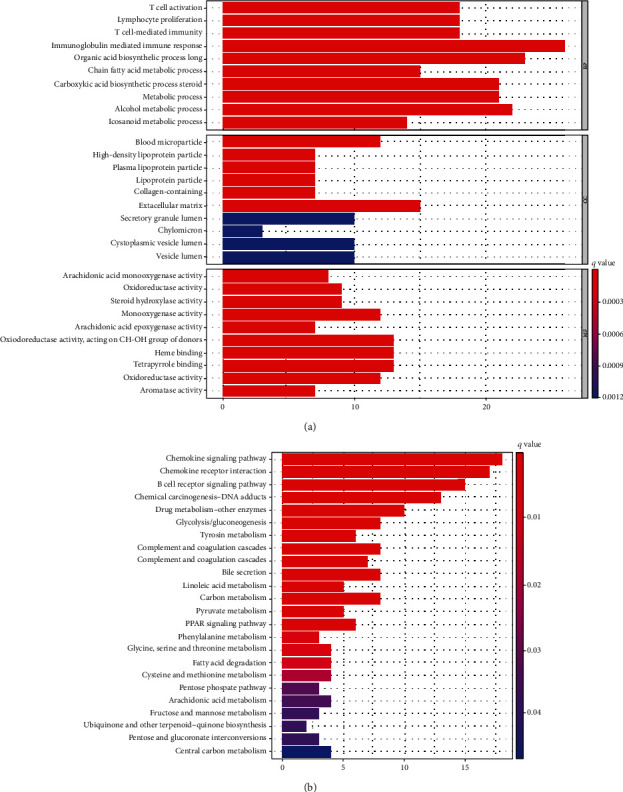
Biological processes and pathways based on risk model. Bar plot graph of (a) GO enrichment and (b) KEGG enrichment for differentially costimulatory molecule genes between both risk groups.

DSMGs (41 raised genes and 94 reduced genes) were chosen, between both groups through “limma” R package (Supplement Table S4). KEGG and GO pathway analysis was implemented to investigate the association between the risk model and the biological process of HCC. The DSMGs were most enriched in biological processes, especially for the activation of T cell, T cell-mediated immunity, lymphocyte proliferation, and immunoglobulin-mediated immune response (Figure 6(a)). The results of KEGG analysis demonstrated that these DSMGs were related to the B cell receptor and chemokine signaling pathway and cytokine receptor interaction (Figure 6(b)).

### 3.6. The Association of Immune Microenvironment and Immune Infiltration with Risk Model

**Figure 7:**
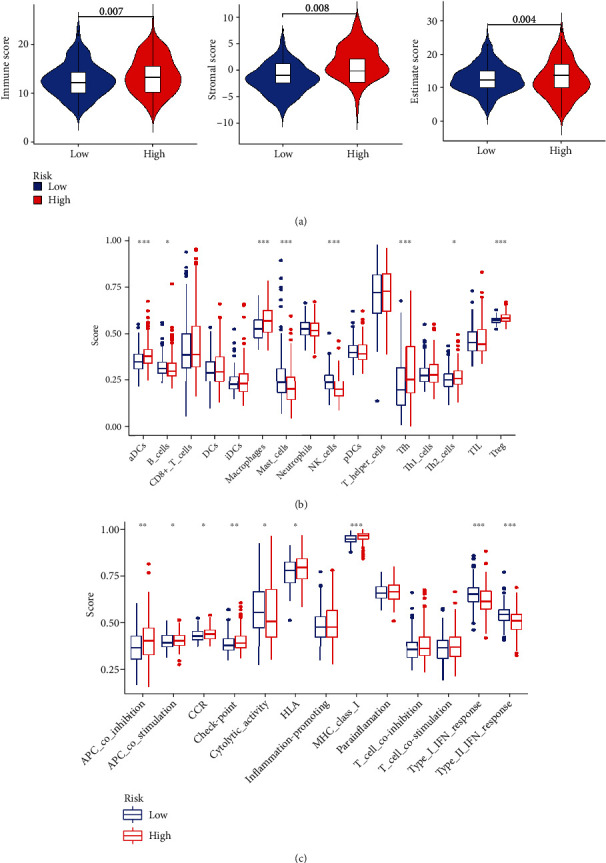
The comparison of immune microenvironment and immune infiltration in both groups. (a) The violin plots demonstrated the differences of estimate score, stromal score, and immune score. The comparison of enrichment score of (b) 16 kinds of immune cells and (c) 13 immune-related pathways between two groups.

Costimulatory molecules affected the activation of T cell, proliferation, and survival, as well as regulated tumor immunity. The high-risk group has higher estimate scores, stromal scores, and immune scores (Figure 7(a)). There existed evident differences in several important immune cells (macrophages, B cells, aDCs, NK cells, mast cells, Treg, Th2, and Tfh cells) between both groups (Figure 7(b)). Besides, the high-risk score group had higher scores of CCR, APC costimulation, APC cosuppression, HLA and MHC class, and checkpoint, while the low-risk score group had higher scores of type II IFN response, type I IFN response, and cytolytic activity (Figure 7(c)).

### 3.7. Immune Therapy and Immune Escape between Two Groups

**Figure 8:**
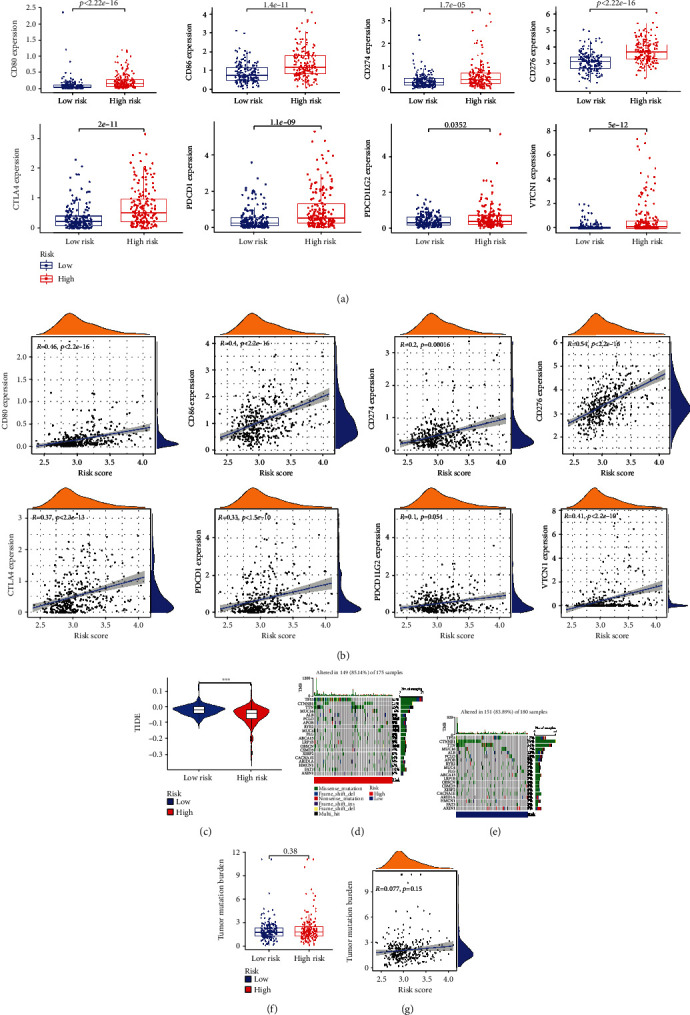
Immune therapy and immune escape between two groups. (a) The box plot demonstrated the comparison of the expressions of eight immune checkpoints molecules. (b) The association of risk score and the expressions of eight immune checkpoint molecules. (c) Comparison of TIDE score between two groups. Waterfall of the first twenty mutated genes in the (d) high- and (e) low-risk groups. (f) Comparison of tumor mutation burden between two groups. (g) The correlation between risk score and tumor mutation burden.

Immune checkpoints are suppressive pathways of the immune system, whose function is an important cause of many diseases. We then explore the expressions of eight immune checkpoint molecules (CD80, CD86, CD274, CD276, CTLA4, PDCD1, PDCD1LG2, and VTCN1) between two groups. Besides PDCD1LG2, the expressions of seven immune checkpoint molecules were higher in the high-risk group (Figure 8(a)), and Pearson analysis showed seven immune checkpoint molecule expression was positively related to risk scores with statistical significance, except PDCD1LG2 (*P* = 0.054), as shown in Figure 8(b). The immune therapeutic effect was evaluated by using TIDE score. The TIDE score in the high-risk group was lower, indicating that immunotherapy deficiency was associated with a dismal prognosis (Figure 8(c)).

### 3.8. Tumor Mutation Burden in Two Groups

The tumor mutation burden scores in two groups were calculated to further explore the effect of immune checkpoint inhibitor therapy. As shown in Figures 8(d) and 8(e), roughly equal mutation events occurred in samples from two groups (85.14% vs. 83.89%), and TP53 was the predominant mutation gene in the two groups. Between both groups, there existed no statistical difference in the samples (Figure 8(f)). Likewise, the tumor mutation burden score and the risk score had no linear correlation (Figure 8(g)).

### 3.9. TNFRSF4 Was an Oncogene in HCC

**Figure 9:**
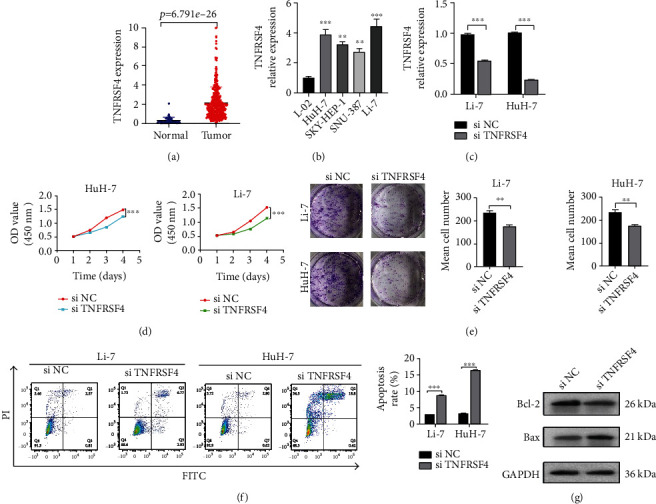
TNFRSF4 was an oncogene in HCC. (a) TNFRSF4 was highly expressed in tumor than normal specimen. (b) The TNFRSF4 expression level in human normal liver and HCC cell lines with qRT-PCR. (c) Validation of siRNA knockdown efficiency in HuH7 and Li-7 cells by qRT-PCR. (d, e) Cell viability of HuH7 and Li-7 cells after knocking down TNFRSF4 was detected using CCK-8 assay and colony assay. (f) Flow cytometry analysis of apoptosis in siNC and siTNFRSF4 transfected HuH7 and Li-7 cells. (g) The relative protein expression of apoptosis was determined in TNFRSF4-knockdown HuH7 cells by using western blot analysis.

For in-depth analysis of the mechanism of our risk model, we selected TNFRSF4 from 8 molecules which constituted the risk model for in-depth investigation. The expressions of TNFRSF4 were higher in HCC from TCGA data (Figure 9(a)). We detected TNFRSF4 expression in one human normal liver and four HCC cell lines and found that the expression level of TNFRSF4 was higher in HCC cell lines with qRT-PCR (Figure 9(b)). For further investigating the TNFRSF4 mechanism, we chose Li-7 and HuH7 for further experiments, which had higher TNFRSF4 expression levels. We transfected siRNA specific for TNFRSF4 in Li-7 and HuH7 cells and detected transfection efficiency by using qRT-PCR (Figure 9(c)). Knocking down TNFRSF4 expression significantly reduced the proliferation ability in Li-7 and HuH7 cell in CCK-8 assays and colon assays (Figures 9(d) and 9(e)). Flow cytometry analysis showed increased apoptosis ability was showed in Li-7 and HuH7 cell, which were transfected with siTNFRSF4 (Figure 9(f)). In addition, upregulation of Bax protein and downregulation of Bcl-2 protein were observed in TNFRSF4-knockdown HuH7 cells (Figure 9(g)).

## 4. Discussion

Targeted therapy with immune checkpoints acted a critical role in cancer immunotherapy [10]. The primary T cell activation needs a synergistic effect of both signals. The first signal is produced with a T cell receptor (TCR) that recognizes a peptide-loaded major histocompatibility complex (pMHC) presented through an antigen-presenting cell (APC) to activate the T cells primarily. The other signal also known as costimulatory molecules was produced by the interaction of the costimulatory molecules on the surface of the APC with the corresponding costimulatory molecules to fully activate T cell [7]. The lack of costimulatory signals is one of the important reasons why tumor cells evade surveillance by the body's immune system [11]. PD1 and cytotoxic T lymphocyte antigen 4 (CTLA4), both members of the CD28 family, are the focus of current immunotherapy research [12]. The costimulatory molecule had been used for the prediction of immunotherapy response and prognosis in several tumors [13, 14]. Nonetheless, the value of costimulatory molecules in the prognosis and immunotherapy in HCC has not been fully explored.

In our research, 17 DSMGs, of which 16 genes were upregulated and one gene was downregulated, were chosen through by comparison between normal tissues and tumor tissues. The “Consensus Cluster Plus” algorithm has been widely used in cancer genomics, where new molecular subclasses of the disease have been identified. On the basis of the costimulatory molecule features, patients with HCC were distributed into two clusters. Statistical differences in survival were shown between the two clusters, indicating expression of the costimulatory molecule gene was correlated with survival.

Then, the costimulatory molecule differential genes were further used to select the prognostic genes. We conducted Cox regression and LASSO regression method and selected eight target genes, including LTBR, RELT, TMIGD2, TNFRSF11A, TNFRSF11B, TNFRSF21, TNFRSF4, and TNFSF4. In accordance with median risk score, HCC patients which come from GEO and TCGA databases were distributed into the low- and high-risk groups. In such databases, the high-risk group exhibited low prognosis. For ROC curve, from GEO and TCGA database, the AUC for the 1-, 2-, and 3-year survival was 0.779, 0.681, and 0.752 and 0.801, 0.751, and 0.720, respectively, which presented the better sensitivity and specificity. Our risk model classified our patients as two diverse areas in t-SNE and PCA analysis, exhibiting outstanding discriminative ability. Additionally, based on the multivariable and univariate Cox regression analysis, the risk score was an essential predictor related to the poor prognosis in GEO and TCGA.

Despite the fact that immunotherapy could improve the prognosis of patients with advanced HCC, only a portion of patients received profit [2]. It had been reported that the effect of tumor immunotherapy was influenced by tumor microenvironment and immune infiltration [15]. Our outcome exhibited that the estimate scores, stromal score, and immune score were higher in the high-risk group. Furthermore, there existed evident differences in several significant immune cells (containing macrophages, B cells, aDCs, NK cells, mast cells, Treg, Th2, and Tfh cells) between both groups, which means that NK cells, mast cells, and B cells acted as an antitumor agent. Recently, one research found that intratumor B cells are thought to be a predictor of improved patient survival and could significantly influence the antitumor immune response [16]. Mast cells made a significant impact on the tumor microenvironment and tumor progression by influencing cell proliferation, angiogenesis, invasion, and metastasis [17]. Natural killer cell (NK cell) which had both cytotoxic and immunomodulatory functions had become a valuable instrument in cancer immunotherapy [18]. Our study also demonstrated that the high-risk score group had higher scores of CCR, APC costimulation, APC cosuppression, HLA and MHC class, and checkpoint, while the low-risk score group had higher scores of type II IFN response, type I IFN response, and cytolytic activity. Those results could bring potential value to targeted therapy for HCC.

The advent of ICIs offered a new and effective treatment for HCC, and drugs represented by PD-1 and PDL-1 have been approved for clinical practice [19]. However, not all patients could benefit from ICIs, the best indication of which remained controversial. Our study demonstrated that seven immune checkpoints molecules (CD80, CD86, CD274, CD276, CTLA4, PDCD1, and VTCN1) were most significantly positively associated with risk scores with statistical significance, which indicated that costimulatory molecule signature could be conducted to evaluate effect of ICB therapy. TIDE score was performed to evaluated effect of ICI therapy between two groups. The score of TIDE was higher in the low-risk group, which may explain the better prognosis. One study showed that TMB was correlated with better overall survival after immunotherapy for a variety of cancer types, suggesting that TMB can be utilized as a predictive biomarker for the therapeutic effect of immune checkpoint inhibitors [20]. Regretfully, the mutation rates of the two groups of patients were 85.14% vs. 83.89%, respectively, with no significant difference. It indicated that the costimulatory molecule was not associated with TMB.

Among eight costimulatory molecules which constructed a prognostic risk model, TNFRSF4 was selected due to the highest coefficient score. TNFRSF4, also named OX40, belonged to the tumor necrosis factor receptor superfamily (TNFRSF). After T cells were costimulated by TNFRSF4, the intracytoplasmic pathways correlated with T cells were activated, like Bcl-2 antiapoptotic molecules, cyclin-dependent kinases, and cyclin A [21]. It had been reported that polymorphism characteristic of TNFRSF4 was related to systemic lupus erythematosus and Sjogren's syndrome [22, 23]. Li et al. [24] reported that TNFSF4 facilitates the cisplatin resistance and suppresses apoptosis of lung adenocarcinoma cells. Our results showed that knocking down TNFRSF4 substantially reduced proliferation ability and increased the apoptosis ability. The detailed molecular mechanisms of TNFSF4 as a carcinogenic factor in HCC deserved further exploration.

Our research inevitably had some limitations. Firstly, TCGA database was utilized to establish the prognostic risk model and only validated it with the GEO database. It is necessary to perform prospective clinical research to examine the predictive and discriminative ability of this model. Secondly, our study demonstrated that immune checkpoint molecule (CD274) was positively associated with risk scores with statistical significance. However, the predictive value of this model for patients who received PD-1 inhibitors therapy was required to be further validated in real word. Thirdly, in vitro experiments confirmed the TNFRSF4 oncogenic effect in HCC, and the mechanism as a tumor promoter needs to be explored in further research.

## 5. Conclusion

A novel risk signature based on eight costimulatory molecules associated with prognosis was constructed to explore the association with survival outcome and immune landscape in patients of HCC. This signature could effectively discriminate patients and accurately predict prognosis. The association of this costimulatory signature with the immune landscape provided an important basis for further research. Besides, our signature could potentially predict response to ICB therapy. The hub costimulatory molecule TNFRSF4 proved to be associated with prognosis and as an oncogene in in vitro experiments.

## Figures and Tables

**Table 1:** The clinicopathological characteristics of patients with HCC from TCGA and GEO database.

Characteristics		TCGA (*n* = 240)	GEO (*n* = 114)
Age		57.12 ± 13.24	63.52 ± 12.72
Gender	Male	165 (68.7%)	93 (81.5%)
	Female	75 (31.2%)	21 (18.4%)
Grade	I	29 (12.0%)	
	II	53 (22.0%)	
	III	95 (39.5%)	
	IV	11 (4.5%)	
TNM stage	I		55 (48.2%)
	II		35 (30.7%)
	III		21 (18.4%)
	IV		3 (2.6%)
T stage	I	118 (49.1%)	
	II	53 (22.0%)	
	III	59 (24.5%)	
	IV	10 (4.1%)	
M stage	M0	236 (98.3%)	
	M1	4 (1.6%)	
N stage	N0	236 (98.3%)	
	N1	4 (1.6%)	
BCLC stage	0		4 (3.5%)
	1		73 (64.0%)
	2		28 (24.5%)
	3		9 (7.8%)

## Data Availability

Data utilized for supporting the results of this work are included in this article and supplementary information.

## Supplementary Materials

Supplementary 1 Supplementary Table 1: the characteristics of the costimulatory molecule genes from previous research.

Supplementary 2 Supplementary Table 2: the qPCR primer sequence was listed.

Supplementary 3 Supplementary Table 3: 10 prognostic costimulatory molecule genes selected by univariate Cox regression analysis for further analysis.

Supplementary 4 Supplementary Table 4: differentially costimulatory molecule genes were selected between the high-risk group and the low-risk group.
