# Supplementary material for: Development of a Costimulatory Molecule Signature to Predict Prognosis, Immune Landscape, and Response to Immune Therapy for Hepatocellular Carcinoma
Source: Dis Markers. 2022 Sep 12;2022:8973721. doi: 10.1155/2022/8973721 (PMC9485710; doi:10.1155/2022/8973721)
Supplement: Supplementary 1 — Supplementary Table 1: the characteristics of the costimulatory molecule genes from previous research. [file 8973721.f1.docx]

| Official symbol | Aliases | Family |
| --- | --- | --- |
| CD27 | TNFRSF7 | TNFRSF |
| CD274 | PD-L1,B7-H1 | B7 |
| CD276 | B7-H3 | B7 |
| CD28 | Tp44 | CD28 |
| CD40 | TNFRSF5 | TNFRSF |
| CD40LG | TNFSF5, CD154, CD4OL | TNFSF |
| CD70 | TNFSF7, CD27L | TNFSF |
| CD80 | B7-1, CD28LG1 | B7 |
| CD86 | B7-2, CD28LG2 | B7 |
| CTLA4 | CD152 | CD28 |
| EDA | EDA-A1, EDA-A2 | TNFSF |
| EDA2R | TNFRSF27, XEDAR | TNFRSF |
| EDAR | EDA-A1R | TNFRSF |
| FAS | TNFRSF6, CD95 | TNFRSF |
| FASLG | TNFSF6, CD95-L | TNFSF |
| HHLA2 | B7-H5 | B7 |
| ICOS | CD278, CVID1 | CD28 |
| ICOSLG | B7-H2. CD275 | B7 |
| LTA | TNFSF1 | TNFSF |
| LTB | TNFSF3 | TNFSF |
| LTBR | TNFRSF3 | TNFRSF |
| NGFR | TNFRSF16, CD271 | TNFRSF |
| PDCD1 | PD-1, CD279 | CD28 |
| PDCD1LG2 | PD-L2,B7DC, CD273 | B7 |
| RELT | TNFRSF19L TNFSF2, TNFA | TNFRSF |
| TMIGD2 | CD28H | CD28 |
| TNF | TNFSF2, TNFA | TNFSF |
| TNFRSF10A | TRAILR1, CD261 | TNFRSF |
| TNFRSF10B | TRAlILR2. CD262 | TNFRSF |
| TNFRSF10C | TRAILR3, CD263 | TNFRSF |
| TNFRSF10D | TRAILR4, CD264 | TNFRSF |
| TNFRSF11A | RANK, CD265 | TNFRSF |
| TNFRSF11B | OPG | TNFRSF |
| TNFRSF12A | FN14, TWEAKR, CD266 | TNFRSF |
| TNFRSF138 | TACI, TNFRSF14B, CD267 | TNFRSF |
| TNFRSF13C | BAFFR, CD268 | TNFRSF |
| TNFRSF14 | LIGHTR. HVEM, CD270 | TNFRSF |
| TNFRSF17 | BCMA, TNFRSF13A, CD269 | TNFRSF |
| TNFRSF18 | GITR, AITR, CD357 | TNFRSF |
| TNFRSF19 | TROY, TAJ | TNFRSF |
| TNFRSF1A | TNFR1, CD120A | TNFRSF |
| TNFRSF1B | TNFR2, CD120B | TNFRSF |
| TNFRSF21 | DR6, CD358 | TNFRSF |
| TNFRSF25 | DR3, TNFRSF12 | TNFRSF |
| TNFRSF4 | 0X40, CD134 | TNFRSF |
| TNFRSF6B | DCR3 | TNFRSF |
| TNFRSF8 | CD30 | TNFRSF |
| TNFRSF9 | 4-1BB, CD137, ILA | TNFRSF |
| TNFSF10 | TRAIL, CD253 | TNFSF |
| TNFSF11 | RANKL, CD254 | TNFSF |
| TNFSF12 | TWEAK | TNFSF |
| TNFSF13 | APRIL, CD256 | TNFSF |
| TNFSF13B | BAFF. CD257 | TNFSF |
| TNFSF14 | LIGHT, HVEML, CD258 | TNFSF |
| TNFSF15 | TL1A | TNFSF |
| TNFSF18 | GITRL | TNFSF |
| TNFSF4 | 0X-40L, CD134L, CD252 | TNFSF |
| TNFSF8 | CD30L, CD153 | TNFSF |
| TNFSF9 | 4-1BB-L, CD137L | TNFSF |
| VTCN1 | B7-H4 | B7 |
