# Supplementary material for: Development of a Costimulatory Molecule Signature to Predict Prognosis, Immune Landscape, and Response to Immune Therapy for Hepatocellular Carcinoma
Source: Dis Markers. 2022 Sep 12;2022:8973721. doi: 10.1155/2022/8973721 (PMC9485710; doi:10.1155/2022/8973721)
Supplement: Supplementary 2 — Supplementary Table 2: the qPCR primer sequence was listed. [file 8973721.f2.docx]

| Name | Sequence (5'->3') |
| --- | --- |
| For qRT- PCR |  |
| TNFRSF4 |  |
| Forward primer | CAGGGACCCTCCACCCG |
| Reverse primer | GAAACTGCCTCCCCCAGG |
| GADPH |  |
| Forward primer | ATTCCATGGCACCGTCAAGG |
| Reverse primer | TGGACTCCACGACGTACTCA |
| siRNAs |  |
| si NC | UUUGUACUACACAAAAGUACUG |
| si HTNFRSF4 | GCACGUGGUGUAACCUCAGAATT |
