# Supplementary material for: Development of a Costimulatory Molecule Signature to Predict Prognosis, Immune Landscape, and Response to Immune Therapy for Hepatocellular Carcinoma
Source: Dis Markers. 2022 Sep 12;2022:8973721. doi: 10.1155/2022/8973721 (PMC9485710; doi:10.1155/2022/8973721)
Supplement: Supplementary 3 — Supplementary Table 3: 10 prognostic costimulatory molecule genes selected by univariate Cox regression analysis for further analysis. [file 8973721.f3.docx]

| id | HR | HR.95L | HR.95H | p-value |
| --- | --- | --- | --- | --- |
| CD276 | 1.33231517 | 1.021689549 | 1.737380707 | 0.034141915 |
| LTBR | 1.522510068 | 1.068210505 | 2.170018828 | 0.020076781 |
| RELT | 2.355287165 | 1.431731826 | 3.874592661 | 0.000743414 |
| TMIGD2 | 0.338115354 | 0.138695241 | 0.824267591 | 0.017077574 |
| TNFRSF11A | 2.080130861 | 1.432484419 | 3.020587411 | 0.000118884 |
| TNFRSF11B | 1.243961245 | 1.067615365 | 1.44943547 | 0.005129293 |
| TNFRSF12A | 1.164866415 | 1.0002671 | 1.35655143 | 0.049599717 |
| TNFRSF21 | 1.205075033 | 1.045883539 | 1.388496693 | 0.009863615 |
| TNFRSF4 | 1.370296256 | 1.060832256 | 1.770036515 | 0.015859485 |
| TNFSF4 | 1.396494946 | 1.094152624 | 1.782382173 | 0.007301167 |
