# Supplementary material for: Development of a Costimulatory Molecule Signature to Predict Prognosis, Immune Landscape, and Response to Immune Therapy for Hepatocellular Carcinoma
Source: Dis Markers. 2022 Sep 12;2022:8973721. doi: 10.1155/2022/8973721 (PMC9485710; doi:10.1155/2022/8973721)
Supplement: Supplementary 4 — Supplementary Table 4: differentially costimulatory molecule genes were selected between the high-risk group and the low-risk group. [file 8973721.f4.docx]

| gene | lowMean | highMean | logFC | pValue | fdr |
| --- | --- | --- | --- | --- | --- |
| DCXR | 9.262521252 | 8.158789198 | -1.103732055 | 1.13E-12 | 1.19E-11 |
| CCL20 | 4.563464262 | 5.72103241 | 1.157568148 | 1.82E-07 | 5.82E-07 |
| S100A6 | 5.742159504 | 6.877104975 | 1.134945471 | 1.08E-07 | 3.60E-07 |
| CYP2A7 | 4.092946213 | 2.888276829 | -1.204669383 | 2.17E-07 | 6.84E-07 |
| PAFAH1B3 | 4.100210771 | 5.129415087 | 1.029204316 | 1.50E-14 | 2.70E-13 |
| FOXQ1 | 2.575398657 | 3.580542042 | 1.005143385 | 1.36E-08 | 5.51E-08 |
| SFN | 3.721446828 | 4.904736389 | 1.18328956 | 3.90E-09 | 1.79E-08 |
| MT1X | 6.905711514 | 5.63789431 | -1.267817204 | 1.36E-06 | 3.65E-06 |
| CYP3A4 | 6.866629789 | 4.953140717 | -1.913489071 | 3.71E-07 | 1.11E-06 |
| PON1 | 7.600311363 | 6.411009898 | -1.189301465 | 1.88E-10 | 1.15E-09 |
| APOA5 | 7.526961179 | 6.157337722 | -1.369623457 | 1.96E-09 | 9.53E-09 |
| GYS2 | 4.471537075 | 3.310701731 | -1.160835344 | 8.02E-13 | 8.69E-12 |
| LOXL4 | 3.122186495 | 4.210992276 | 1.088805781 | 1.15E-06 | 3.12E-06 |
| SPINK1 | 6.063517631 | 7.202365266 | 1.138847635 | 0.005991244 | 0.008647351 |
| HPX | 10.26163209 | 8.955491938 | -1.306140151 | 5.46E-18 | 3.34E-16 |
| SLC16A3 | 2.618056143 | 3.620060673 | 1.00200453 | 3.47E-20 | 6.76E-18 |
| RTP3 | 5.512941431 | 4.261960868 | -1.250980563 | 1.73E-08 | 6.82E-08 |
| UBE2C | 3.823871914 | 5.002126843 | 1.178254929 | 6.40E-16 | 1.78E-14 |
| RBP4 | 12.16636773 | 11.07224369 | -1.094124047 | 2.04E-18 | 1.42E-16 |
| A1BG | 5.187961085 | 4.174064544 | -1.013896541 | 1.39E-14 | 2.52E-13 |
| GLYATL1 | 4.977513295 | 3.796565383 | -1.180947912 | 3.19E-16 | 9.78E-15 |
| FTCD | 7.547225254 | 6.389382772 | -1.157842482 | 4.53E-15 | 9.50E-14 |
| DKK1 | 2.177037774 | 3.183891459 | 1.006853686 | 7.85E-07 | 2.20E-06 |
| IER3 | 4.70281261 | 5.816238522 | 1.113425912 | 3.18E-11 | 2.34E-10 |
| UGT1A4 | 4.809227904 | 3.770615338 | -1.038612566 | 5.04E-06 | 1.22E-05 |
| SLC1A5 | 3.044107028 | 4.226321595 | 1.182214566 | 1.54E-19 | 1.95E-17 |
| SLC10A1 | 7.054445536 | 5.201529421 | -1.852916115 | 1.54E-13 | 2.03E-12 |
| CDO1 | 7.64531572 | 6.445309949 | -1.200005771 | 5.42E-14 | 8.10E-13 |
| CYP2E1 | 8.116386305 | 6.307859898 | -1.808526407 | 8.91E-07 | 2.47E-06 |
| SAA4 | 8.088559919 | 6.682816288 | -1.405743631 | 8.50E-11 | 5.63E-10 |
| HPD | 9.164056561 | 6.979776934 | -2.184279627 | 4.37E-12 | 3.96E-11 |
| CES2 | 7.873044405 | 6.779107311 | -1.093937094 | 3.33E-13 | 3.94E-12 |
| CYP2B6 | 4.955009647 | 3.937789167 | -1.01722048 | 1.20E-08 | 4.96E-08 |
| HSD17B6 | 8.64331996 | 7.358197268 | -1.285122692 | 2.33E-12 | 2.25E-11 |
| TTC36 | 4.238970117 | 2.732091207 | -1.50687891 | 1.30E-15 | 3.23E-14 |
| S100A11 | 5.921247643 | 7.015322625 | 1.094074982 | 1.53E-13 | 2.02E-12 |
| MMP7 | 2.145227343 | 3.322859214 | 1.17763187 | 3.45E-09 | 1.60E-08 |
| TAT | 7.908275014 | 5.915893325 | -1.992381688 | 1.22E-13 | 1.65E-12 |
| ALDH1L1 | 6.375473589 | 5.265712523 | -1.109761066 | 1.32E-05 | 2.97E-05 |
| PGC | 2.097955198 | 3.144076438 | 1.04612124 | 1.31E-08 | 5.33E-08 |
| PLG | 8.712612983 | 7.663307214 | -1.049305769 | 5.71E-09 | 2.52E-08 |
| GSTM1 | 4.136609253 | 3.039278318 | -1.097330936 | 0.00547911 | 0.007945693 |
| FETUB | 6.285356466 | 4.960968685 | -1.324387781 | 4.39E-09 | 1.99E-08 |
| UROC1 | 4.044985176 | 3.036056384 | -1.008928793 | 2.56E-08 | 9.75E-08 |
| DNASE1L3 | 4.230353699 | 2.867718295 | -1.362635404 | 1.33E-17 | 6.87E-16 |
| TTR | 10.64461849 | 9.50222656 | -1.142391931 | 7.02E-08 | 2.43E-07 |
| RDH16 | 6.587793271 | 5.293907879 | -1.293885391 | 1.64E-09 | 8.13E-09 |
| C8A | 7.767135135 | 6.753662405 | -1.01347273 | 1.45E-10 | 9.11E-10 |
| CYP4A11 | 7.76778253 | 6.568379219 | -1.199403311 | 2.66E-09 | 1.25E-08 |
| SPP1 | 5.097804679 | 7.702042559 | 2.60423788 | 5.54E-14 | 8.24E-13 |
| SLC27A5 | 7.005590928 | 5.39319969 | -1.612391238 | 5.44E-19 | 5.01E-17 |
| PCK1 | 7.096230653 | 5.571253852 | -1.524976801 | 2.14E-10 | 1.29E-09 |
| CDC20 | 3.593499737 | 5.043895362 | 1.450395625 | 2.91E-19 | 3.04E-17 |
| GAL3ST1 | 2.580518335 | 3.663241425 | 1.08272309 | 6.35E-12 | 5.51E-11 |
| CYP1A2 | 4.264217434 | 3.105970351 | -1.158247083 | 4.20E-05 | 8.65E-05 |
| FBP1 | 8.260314678 | 6.997494566 | -1.262820111 | 3.98E-13 | 4.64E-12 |
| COL1A1 | 4.445500709 | 5.638159166 | 1.192658457 | 6.13E-06 | 1.46E-05 |
| HGFAC | 5.176909602 | 3.743806527 | -1.433103075 | 2.98E-06 | 7.52E-06 |
| ADH4 | 8.258888963 | 6.444506839 | -1.814382124 | 5.10E-10 | 2.83E-09 |
| COX7B2 | 2.383133363 | 3.414037596 | 1.030904233 | 0.000179868 | 0.000333017 |
| MMP9 | 2.89840929 | 4.011990976 | 1.113581686 | 1.05E-11 | 8.69E-11 |
| SLC22A1 | 6.418440211 | 4.810475394 | -1.607964817 | 1.65E-09 | 8.17E-09 |
| TOP2A | 3.607080259 | 4.875009057 | 1.267928797 | 3.41E-18 | 2.19E-16 |
| APOA1 | 12.54425865 | 11.3868223 | -1.157436357 | 4.11E-09 | 1.88E-08 |
| CYP2C8 | 7.749063014 | 6.314100008 | -1.434963006 | 2.99E-10 | 1.74E-09 |
| INSIG1 | 8.155390957 | 7.136924827 | -1.01846613 | 1.30E-09 | 6.62E-09 |
| ALDOB | 11.10450924 | 9.604798723 | -1.499710513 | 9.09E-11 | 5.98E-10 |
| SPP2 | 6.827367529 | 5.293768813 | -1.533598716 | 6.26E-10 | 3.41E-09 |
| GNMT | 6.497203232 | 5.01039541 | -1.486807822 | 4.56E-12 | 4.11E-11 |
| FNDC5 | 4.181336743 | 3.174628648 | -1.006708095 | 5.25E-06 | 1.27E-05 |
| HMGA1 | 4.994860368 | 6.011902735 | 1.017042367 | 2.23E-19 | 2.52E-17 |
| ACSM2A | 5.939691425 | 4.755535379 | -1.184156046 | 5.23E-13 | 5.90E-12 |
| HRG | 9.220775793 | 7.932516659 | -1.288259134 | 3.49E-07 | 1.05E-06 |
| SDS | 6.87167627 | 5.421894595 | -1.449781674 | 5.50E-06 | 1.32E-05 |
| CFHR3 | 5.381544115 | 4.277615602 | -1.103928513 | 4.21E-08 | 1.52E-07 |
| HP | 11.10573667 | 9.862326502 | -1.243410165 | 1.95E-09 | 9.48E-09 |
| F12 | 8.691400589 | 7.571765735 | -1.119634854 | 1.03E-10 | 6.67E-10 |
| BHMT | 7.355191276 | 6.187105814 | -1.168085463 | 1.91E-07 | 6.07E-07 |
| GLYAT | 5.296113934 | 3.908282532 | -1.387831402 | 1.23E-11 | 9.98E-11 |
| AGXT | 9.265705967 | 8.103117925 | -1.162588041 | 1.83E-10 | 1.12E-09 |
| HSD17B13 | 5.085627378 | 4.025003622 | -1.060623756 | 0.000196997 | 0.000361923 |
| TPX2 | 3.552666114 | 4.593525959 | 1.040859845 | 2.32E-19 | 2.57E-17 |
| AQP9 | 7.875634837 | 6.497400281 | -1.378234556 | 6.48E-07 | 1.85E-06 |
| APOC3 | 12.62246183 | 11.32768055 | -1.294781279 | 1.84E-12 | 1.82E-11 |
| AFP | 3.659176793 | 5.236870817 | 1.577694023 | 2.13E-05 | 4.61E-05 |
| CPS1 | 8.300583077 | 6.784387816 | -1.516195261 | 2.08E-09 | 1.00E-08 |
| MCM2 | 3.509606064 | 4.519315983 | 1.009709918 | 9.90E-17 | 3.59E-15 |
| RAP1GAP | 3.822771189 | 4.941411297 | 1.118640108 | 2.01E-15 | 4.79E-14 |
| PRAP1 | 8.706011583 | 7.695211969 | -1.010799614 | 1.49E-06 | 3.97E-06 |
| OTC | 7.161168376 | 5.913202665 | -1.247965711 | 1.84E-09 | 9.02E-09 |
| HPR | 8.194418498 | 6.691303358 | -1.50311514 | 6.93E-11 | 4.68E-10 |
| CYP2C9 | 7.516391597 | 6.079935876 | -1.436455721 | 5.67E-12 | 4.97E-11 |
| CD24 | 4.724678239 | 6.432307444 | 1.707629206 | 1.55E-11 | 1.23E-10 |
| SULT2A1 | 8.88927418 | 7.826531902 | -1.062742278 | 3.07E-05 | 6.48E-05 |
| FMO3 | 7.764845294 | 6.680538479 | -1.084306815 | 2.49E-08 | 9.47E-08 |
| TRNP1 | 3.30133814 | 4.574721936 | 1.273383796 | 4.63E-13 | 5.32E-12 |
| LECT2 | 6.160932674 | 4.90566037 | -1.255272304 | 1.40E-07 | 4.56E-07 |
| PON3 | 6.930623127 | 5.88096257 | -1.049660556 | 8.59E-16 | 2.28E-14 |
| SOX9 | 3.450378435 | 4.604825298 | 1.154446863 | 1.58E-14 | 2.81E-13 |
| ACSM2B | 6.305438728 | 5.224219845 | -1.081218882 | 1.27E-10 | 8.08E-10 |
| C6 | 7.177764207 | 5.828839372 | -1.348924835 | 6.41E-15 | 1.29E-13 |
| SPHK1 | 2.362307432 | 3.447160592 | 1.084853161 | 2.87E-16 | 8.94E-15 |
| G6PD | 3.610338096 | 4.899024383 | 1.288686286 | 9.36E-23 | 1.32E-19 |
| MYBL2 | 2.910782323 | 4.184035432 | 1.273253109 | 1.51E-19 | 1.94E-17 |
| F9 | 6.957941758 | 5.581014996 | -1.376926762 | 6.95E-09 | 3.03E-08 |
| ADH1B | 8.213052265 | 6.54281076 | -1.670241505 | 1.69E-11 | 1.33E-10 |
| TDO2 | 5.890802978 | 4.856731234 | -1.034071744 | 2.00E-05 | 4.36E-05 |
| GCGR | 4.597730078 | 3.132558266 | -1.465171813 | 4.79E-09 | 2.15E-08 |
| APOF | 5.914796433 | 4.416517164 | -1.49827927 | 2.19E-10 | 1.32E-09 |
| HKDC1 | 3.624417425 | 4.720474741 | 1.096057316 | 1.78E-09 | 8.76E-09 |
| ADH1A | 8.943256982 | 7.643669362 | -1.29958762 | 2.68E-11 | 1.99E-10 |
| CYP8B1 | 6.90678453 | 5.104765428 | -1.802019102 | 2.94E-11 | 2.17E-10 |
| UPB1 | 6.386625054 | 5.305757481 | -1.080867573 | 4.66E-11 | 3.28E-10 |
| HAO1 | 7.685254584 | 6.584094586 | -1.101159998 | 9.64E-13 | 1.03E-11 |
| SLC38A1 | 2.819444718 | 3.864799261 | 1.045354543 | 8.34E-18 | 4.74E-16 |
| CYP2A6 | 7.257903786 | 5.377630826 | -1.88027296 | 1.42E-08 | 5.73E-08 |
| CCL16 | 6.702126513 | 5.63669947 | -1.065427044 | 1.07E-05 | 2.43E-05 |
| HSD11B1 | 7.272572471 | 6.22987169 | -1.04270078 | 0.000232733 | 0.000422452 |
| DSG2 | 3.435811493 | 4.436574368 | 1.000762875 | 3.28E-14 | 5.29E-13 |
| CYP4A22 | 4.971390449 | 3.917406923 | -1.053983526 | 1.36E-11 | 1.09E-10 |
| GSTA1 | 9.807117812 | 8.420998582 | -1.38611923 | 2.95E-08 | 1.11E-07 |
| AKR7A3 | 6.319576885 | 5.225262137 | -1.094314747 | 6.34E-09 | 2.79E-08 |
| S100A9 | 3.852929603 | 5.131532155 | 1.278602552 | 3.83E-09 | 1.76E-08 |
| S100P | 3.295404431 | 4.386791069 | 1.091386638 | 7.25E-05 | 0.000143534 |
| AFM | 7.419738011 | 5.898916064 | -1.520821948 | 3.88E-11 | 2.79E-10 |
| CA9 | 1.935757158 | 3.284495152 | 1.348737993 | 7.40E-19 | 6.28E-17 |
| ADH1C | 8.340406286 | 6.528922814 | -1.811483473 | 1.25E-10 | 7.95E-10 |
| SLC13A5 | 6.471142637 | 5.092783314 | -1.378359323 | 1.43E-12 | 1.46E-11 |
| SERPINC1 | 11.52465657 | 10.03867822 | -1.485978341 | 1.03E-15 | 2.66E-14 |
| CFHR4 | 4.85582067 | 3.67327945 | -1.18254122 | 2.81E-11 | 2.08E-10 |
| AZGP1 | 8.825479728 | 7.747387938 | -1.07809179 | 7.50E-12 | 6.39E-11 |
| UBD | 6.292617547 | 7.466224632 | 1.173607085 | 1.04E-07 | 3.48E-07 |
| MT2A | 8.725259773 | 7.671683418 | -1.053576355 | 1.93E-05 | 4.23E-05 |
| SOX4 | 3.341063793 | 4.403853425 | 1.062789632 | 2.64E-14 | 4.38E-13 |
| UGT2B10 | 7.599808971 | 6.412705646 | -1.187103324 | 6.13E-08 | 2.15E-07 |
